# Identification of ferroptosis related genes and pathways in prostate cancer cells under erastin exposure

**DOI:** 10.1186/s12894-024-01472-1

**Published:** 2024-04-04

**Authors:** Fan Wu, Fei Huang, Nili Jiang, Jinfeng Su, Siyi Yao, Boying Liang, Wen Li, Tengyue Yan, Sufang Zhou, Qingniao Zhou

**Affiliations:** 1 Department of Biochemistry and Molecular Biology, School of Pre-Clinical Medicine, Guangxi Medical University, Nanning, China; 2 Key Laboratory of Biological Molecular Medicine Research, Education Department of Guangxi Zhuang Autonomous Region, Guangxi Medical University, Nanning, China; 3 Life Sciences Institute, Guangxi Medical University, Nanning, China

**Keywords:** Ferroptosis, Erastin, Prostate cancer, TMEFF2, NRXN3, RNA-seq

## Abstract

**Background:**

Few studies are focusing on the mechanism of erastin acts on prostate cancer (PCa) cells, and essential ferroptosis-related genes (FRGs) that can be PCa therapeutic targets are rarely known.

**Methods:**

In this study, in vitro assays were performed and RNA-sequencing was used to measure the expression of differentially expressed genes (DEGs) in erastin-induced PCa cells. A series of bioinformatic analyses were applied to analyze the pathways and DEGs.

**Results:**

Erastin inhibited the expression of *SLC7A11* and cell survivability in LNCaP and PC3 cells. After treatment with erastin, the concentrations of malondialdehyde (MDA) and Fe^2+^ significantly increased, whereas the glutathione (GSH) and the oxidized glutathione (GSSG) significantly decreased in both cells. A total of 295 overlapping DEGs were identified under erastin exposure and significantly enriched in several pathways, including DNA replication and cell cycle. The percentage of LNCaP and PC3 cells in G1 phase was markedly increased in response to erastin treatment. For four hub FRGs, *TMEFF2* was higher in PCa tissue and the expression levels of *NRXN3*, *CLU*, and *UNC5B* were lower in PCa tissue. The expression levels of *SLC7A11* and cell survivability were inhibited after the knockdown of *TMEFF2* in androgen-dependent cell lines (LNCaP and VCaP) but not in androgen-independent cell lines (PC3 and C4-2). The concentration of Fe^2+^ only significantly increased in *TMEFF2* downregulated LNCaP and VCaP cells.

**Conclusion:**

*TMEFF2* might be likely to develop into a potential ferroptosis target in PCa and this study extends our understanding of the molecular mechanism involved in erastin-affected PCa cells.

**Supplementary Information:**

The online version contains supplementary material available at 10.1186/s12894-024-01472-1.

## Introduction

As a highly lethal cancer, prostate cancer (PCa) ranks second in the incidence of male malignant tumors worldwide [1]. Androgen deprivation therapy is the clinical treatment for PCa, however, patients receiving this treatment may develop castration-resistant prostate cancer (CRPC) [2]. Therefore, it is necessary to screen and identify more targeted genes and develop a more effective therapy for PCa.

As a unique mode of cell death, ferroptosis is widely divergent from other types of cell death, including apoptosis, autophagy, and necrosis [3]. Mechanically, a few essential factors or products of lipid peroxidation metabolism, such as solute carrier family 7 member 11 (SLC7A11), glutathione peroxidase (GPX4), glutathione (GSH), and malondialdehyde (MDA), are related to ferroptosis [4]. Considering that PCa with metastatic potential [5] and cancer cells with metastatic and invasive abilities are susceptible to ferroptosis [6], we hypothesized that targeting certain ferroptosis-related genes (FRGs) may be useful in treating PCa. An increasing number of researchers have focused on the relationship between ferroptosis and PCa. Wo et al. identified some FRGs by analyzing RNA sequencing (RNA-seq) data from The Cancer Genome Atlas (TCGA) [7]. Unfortunately, they only included the datasets from the clinical data of PCa patients and did not analyze the data of PCa cells because of the lack of RNA-seq results derived from ferroptotic PCa cells. However, focusing on ferroptosis in PCa cells will provide a more comprehensive theoretical basis or treatment strategy for clinical treatment. Therefore, we sought to obtain the RNA-seq information to clarify the potential mechanisms of ferroptotic PCa cells.

As one of the most famous ferroptosis inducers, erastin is a perfect drug to trigger the ferroptotic progress of cells [8]. There are few studies and limited information on the impact of erastin on PCa cells. Ghoochani A et al. used cell and animal tests to improve various ferroptosis inducers, including erastin, and remarkably slowed PCa progression [9]. Yang et al. claimed that erastin can downregulate androgen receptor expression in both PCa cells and animal models [10]. After analyzing the data of PCa patients in a public database, Wo et al. found and validated several FRGs [7]. Based on the studies above, we realize that erastin exerts inhibitory effects on PCa cells or tumor growth, and we asked two questions: what are the mechanisms by which erastin acts on PCa cells? Which FRGs play an essential role in ferroptosis triggered by erastin? To answer these questions, erastin was selected as a ferroptosis inducer to construct a ferroptotic PCa cell model. Moreover, PC3 is an androgen-independent cell line, and LNCaP is an androgen-dependent cell line [11], so these two different and representative PCa cell lines were selected.

In our research, cellular experiments were performed in erastin-induced LNCaP and PC3 cells, and the impact of erastin on ferroptotic levels in PCa cells was studied. RNA-seq was applied to screen and identify the differentially expressed genes (DEGs) in PCa cells under erastin exposure. Ferroptosis-related pathways and FRGs that may play an essential role in erastin-induced PCa cell lines were identified. In addition, we tried to provide ferroptotic clues in terms of pathways, transcription factors (TFs), and modules for two different cells. Several FRGs with clinical significance were analyzed and validated. Hence, our study not only extends our understanding of erastin-affected PCa cells but also provides potential therapeutic targets and ideas for PCa therapy.

## Materials and methods

### Cell lines and reagents

LNCaP, PC3, VCaP and C4-2 cells (a gift from the Suzhou Institute of Biomedical Engineering and Technology, Chinese Academy of Sciences) were cultured at 37 °C in RPMI-1640 containing 15% and 10% fetal bovine serum (BI, Biological Industries, Co., Ltd., Israel), respectively. The ferroptosis inducer erastin (HY-15,763) and the androgen receptor (AR) inhibitor enzalutamide (HY-70,002) were purchased from MCE company. A virus expressing a short hairpin RNA (shRNA) targeting *TMEFF2* was obtained from GenePharma Co., Ltd., for infection. The *TMEFF2* targeted shRNA sequence was as follows: 5′-GUGUGAGCAUUCUAUCAAU-3′ [12]. Stably infected cells exhibiting *TMEFF2* knockdown were obtained by selective screening with puromycin (1 µg/mL; Solarbio, China).

### Western blot analysis

For Western blot analysis, proteins isolated from the control cell samples (LNCaP and PC3) and from the experimental cell samples were collected after incubation with 5.0 µM erastin for 24 h. In addition, proteins isolated from the control cell samples (LNCaP and VCaP) and from the experimental cell samples were collected after incubation with 10.0 µM enzalutamide for 48 h. Each protein from these samples was separated in a 10% sodium dodecyl sulfate polyacrylamide gel and transferred to a polyvinylidene difluoride membrane. These membranes were probed with primary antibodies against SLC7A11 (1:250, ab307601, Abcam, USA), TMEFF2 (1:500, ab133562, Abcam, USA), AR (1:500, ab198394, Abcam, USA), and β-tubulin (1:2000, 10094-1-AP, ProteinTech, China). Subsequently, the membranes and the corresponding secondary antibody (ProteinTech, China) were incubated at room temperature for 1 h before being photographed.

### Cell survivability assay

Herein, 2.5 × 10^3^ cells were inoculated in 96-well plates. After 24 h, 5.0 µM erastin was added to the culture medium. Each well was added with CCK-8 solution (Dojindo, Japan) was added to each well at 24 h, 48 h, 72 h, and 96 h. The viability of the cells was determined by measuring the absorbance values of the 96-well plates at 450 nm using a quantitative microplate spectrophotometer (BioTek, Winooski, USA).

### Malondialdehyde (MDA) assay

A micro malondialdehyde assay kit (BC0025, Solarbio, China) was used to detect the cellular MDA levels. After 5 × 10^6^ cells were in the logarithmic growth phase, they were treated with 5 µM erastin for 24 h. These cells were lysed and cracked with extracting solution by using an ultrasonic cell disruptor. The supernatants were obtained after a series of centrifugation and preservation steps at specific temperatures based on the manufacturer’s instructions. Finally, the light absorption values at 532 nm and 600 nm wavelengths of the two groups’ supernatants were detected, calculated, and analyzed.

### Ferrous ion (Fe^2+^) concentration assay

An intracellular iron colorimetric assay kit (E1042, Applygen, China) was used to measure the level of Fe^2+^. After cells were treated with 5 µM erastin for 24 h, the cell lysis solution and a shaker were used for 2 h to lyse a total of 2 × 10^6^ cells. Moreover, the diluted reagents were mixed, incubated, and centrifuged to acquire a standard solution and then subsequently stained with 30 µl Fe^2+^ detection reagent for 30 min. The absorption of the reagent at 550 nm wavelength was measured by a quant microplate spectrophotometer.

### Glutathione (GSH) and oxidized glutathione (GSSG) assay

Herein, the control and experimental group cells after treatment with 5 µM erastin for 24 h were prepared in a 6-well plate, collected and homogenized with 150 µl of protein removal reagent. Then, the samples were placed in liquid nitrogen and a 37 °C water bath twice in sequence to rupture the cells. Afterward, cell samples were placed on ice and then centrifuged to obtain the supernatant. Subsequently, GSH and GSSG assay kits (Beyotime, China) were used to measure and analyze the GSH and GSSG levels, and the absorbance at 412 nm wavelength was measured.

### Sample collection and detection

After washing with PBS, the cells were digested and obtained, which were then sent to Novogene, Co., Ltd., China. 1 µg RNA from cells was extracted by the Illumina TruSeq RNA Sample Prep Kit (FC-122-1001, Illumina, USA) to construct the sequencing libraries. The purified double-stranded cDNA was synthesized by Olygo (dT) reverse transcription, amplified by PCR, and screened with AMPureXP beads to acquire cDNA libraries. RNA-seq was performed on the Illumina NovaSeq 6000 to generate 150 bp paired-end readings, and approximately 8 G of reads per sample were obtained. Data are available in the Gene Expression Omnibus (GEO) database (GEO submission number: GSE232034).

### Data processing

First, sequencing adapters and low-quality reads were trimmed and removed. Then, Homo_sapiens_Ensemble_94 was selected as the reference genome sequence for high-quality reads. The feature counts were applied to calculate the reads and the fragments per kilobase of the exon model per million mapped fragments (FPKM) for the length of genes. Pearson correlation and principal component analyses (PCA) were selected to analyze the sample correlation and sample clustering, respectively. The R (Version 3.0.3) ggplot2 package was used for visualization.

### DEGs analysis

The DEGs analysis was performed within the experimental group (LNCaP_5_0_era and PC3_5_0_era) and the control group (LNCaP and PC3) using DESeq2 software (1.20.0), and each sample group was performed in triplicate. DESeq2 offers statistical procedures to determine the DEGs by the negative binomial distribution model [13]. The significant differential expression mean *p-*value (padj) ≤ 0.05 & |log2(foldchange)| ≥ 2.

### Functional enrichment analysis of erastin-induced genes

For the potential functions and signaling pathways of erastin-induced gene expression changes in PCa cells to be further explored, Gene Ontology (GO), Kyoto Encyclopedia of Genes and Genomes (KEGG), and Reactome analyses were performed to explore and study the different overlapping DEGs of the LNCaP group (LNCaP_5_0_era and LNCaP) and the PC3 group (PC3_5_0_era and PC3).

### Cell cycle analysis

The control group cells and experimental group cells treated with 5 µM erastin treatment for 24 h were prepared in a 6-well plate. Approximately 1 × 10^6^ cells were washed with PBS and centrifuged to obtain the cells. After the cells were incubated with the mixture in the Cell Cycle Analysis Kit (MultiSciences, China), the flow cytometry (BD company, USA) and ModFit LT software were used to analyze the cell cycle distribution.

### Screening of hub genes and module analysis

A protein‒protein interaction (PPI) network obtained through different overlapping DEGs of the LNCaP group (LNCaP_5_0_era and LNCaP) and the PC3 group (PC3_5_0_era and PC3) was submitted to the STRING database [14]. Cytoscape (version 3.9.1) [15] was applied to analyze the PPI relationships, and a total score of > 0.9 was selected as the cutoff criterion. In addition, CytoHubba obtained the top 10 hub genes during the screening process [16]. The Cytoscape plug-in MCODE was employed to detect the molecular complex and acquire the module of DEGs.

### Gene set enrichment analysis (GSEA)

GSEA enrichment analysis [17] was carried out to analyze the GO datasets of the LNCaP group (LNCaP_5_0_era vs. LNCaP) and the PC3 group (PC3_5_0_era vs. PC3) separately. The significant GSEA results with the stringed threshold of nominal *p-*value < 0.05, false discovery rate (FDR) q-value < 0.25, and |normalized enrichment score (NES)| >1.

### Prediction of hub genes’ TFs

A total of 10 hub genes were submitted to the NetworkAnalyst platform to predict their TFs. The putative TFs associated with hub genes were identified and sorted through the mean rank score.

### qPCR

Total RNA was extracted from the control samples (LNCaP and PC3 cells) as well as from the experimental samples (the control cells under erastin treatment) using the TRIzol (Beyotime, China) method. The cDNA was obtained by using the HiScript II 1st Strand cDNA Synthesis Kit (R211-01, Vazyme, China) and qPCR was performed in triplicate using AceQ qPCR SYBR Green Master Mix (Q111-02, Vazyme, China). The expression of glyceraldehyde-phosphate dehydrogenase (GAPDH) was employed to compare with the hub genes and TFs, which were measured with the 2-∆∆CT method. The qPCR primers used in this study are listed in Supplementary Table 1.

### Validation for FRGs

The Human Protein Atlas database was used to determine the gene expression patterns of interested hub genes in tissues. The GEPIA platform was selected to draw a box map of the hub genes with illustrated the expression patterns of tissues [18]. In addition, this online tool provided the correlation analysis for the hub gene. The online database GeneMANIA created an interactive network to predict the tangible relationship between the hub genes [19]. Western blotting, Fe^2+^ concentration assays, and CCK8 assays were performed to assess the correlation between the hub genes and ferroptosis.

The gene expression data of the GSE35988 dataset were downloaded from the public GEO database. We extracted the raw data (including 29 of normal tissue samples, 81of localized prostate cancer samples, and 35 of CRPC samples) from the GPL6848 and GPL6480 platforms. The data from these two platforms were merged and batch removed. The differentially expressed genes were obtained from the analysis through the limma R package (version 3.6.2). The significant differential expression mean *p*-value (padj) ≤ 0.05 & |log2(foldchange)| ≥ 1.

## Results

### Erastin accelerates ferroptosis of PCa cells

**Fig. 1:**
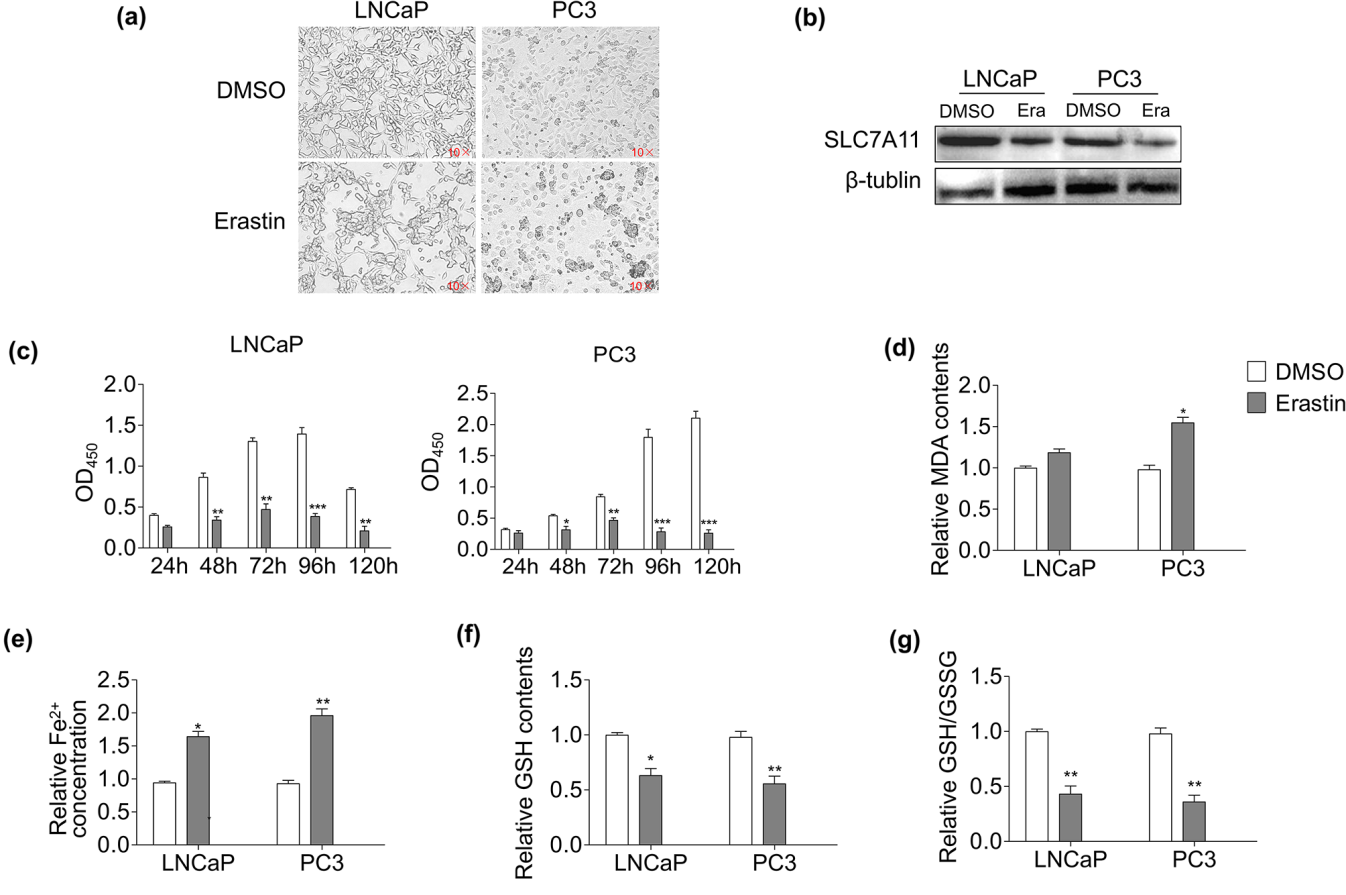
Erastin inhibits cell proliferation and induces ferroptosis in LNCaP and PC3 cells. (**a**) Phenotypic characterization of LNCaP and PC3 groups; “DMSO”, cells induced by DMSO as a negative control; “Erastin”, cells induced by erastin as an experimental group. (**b**) Expression of SLC7A11 was suppressed by erastin treatment in LNCaP and PC3 cells. (**c**) Proliferative levels of LNCaP and PC3 cells after erastin treatment. The relative MDA (**d**), Fe^2+^ (**e**), GSH (**f**), and GSH/GSSG (**g**) levels of LNCaP and PC3 cells treated with erastin were detected. Three independent experiments were performed with triplicate wells. Representative experiments and images are shown. Statistical significance was calculated using an independent-sample t-test. *p < 0.05, **p < 0.01, ***p < 0.001

The LNCaP and PC3 cell lines treated with erastin were phenotypically different in the two cell groups (Fig. 1a). Erastin repressed the expression of the ferroptosis marker protein SLC7A11 in both PCa cell lines (Fig. 1b). Thus, we carried out cell survivability, MDA, Fe^2+^, GSH, and GSSG assays of erastin-induced PCa cells. After 48 h, erastin significantly inhibited the proliferation of LNCaP and PC3 cells in the CCK8 assay (*p* < 0.05; Fig. 1c). The results showed that MDA levels under erastin-induced conditions were notably increased in PC3 cells (*p* < 0.05; Fig. 1d). In contrast, erastin did not trigger a marked difference in MDA levels in LNCaP cells (*p* > 0.05; Fig. 1d). Fe^2+^ content, one of the key features of ferroptosis, was notably increased with erastin treatment in these two cell lines (*p* < 0.05; Fig. 1e). Given that GSH/GSSG regulates cellular redox homeostasis, the levels of GSH and GSSG were measured. The results showed that GSH levels in both cell lines were decreased following erastin treatment (*p* < 0.05; Fig. 1f). Meanwhile, the GSH/GSSG levels were also downregulated in these cell samples (*p* < 0.01; Fig. 1g).

### Summary of RNA sequencing data

**Fig. 2:**
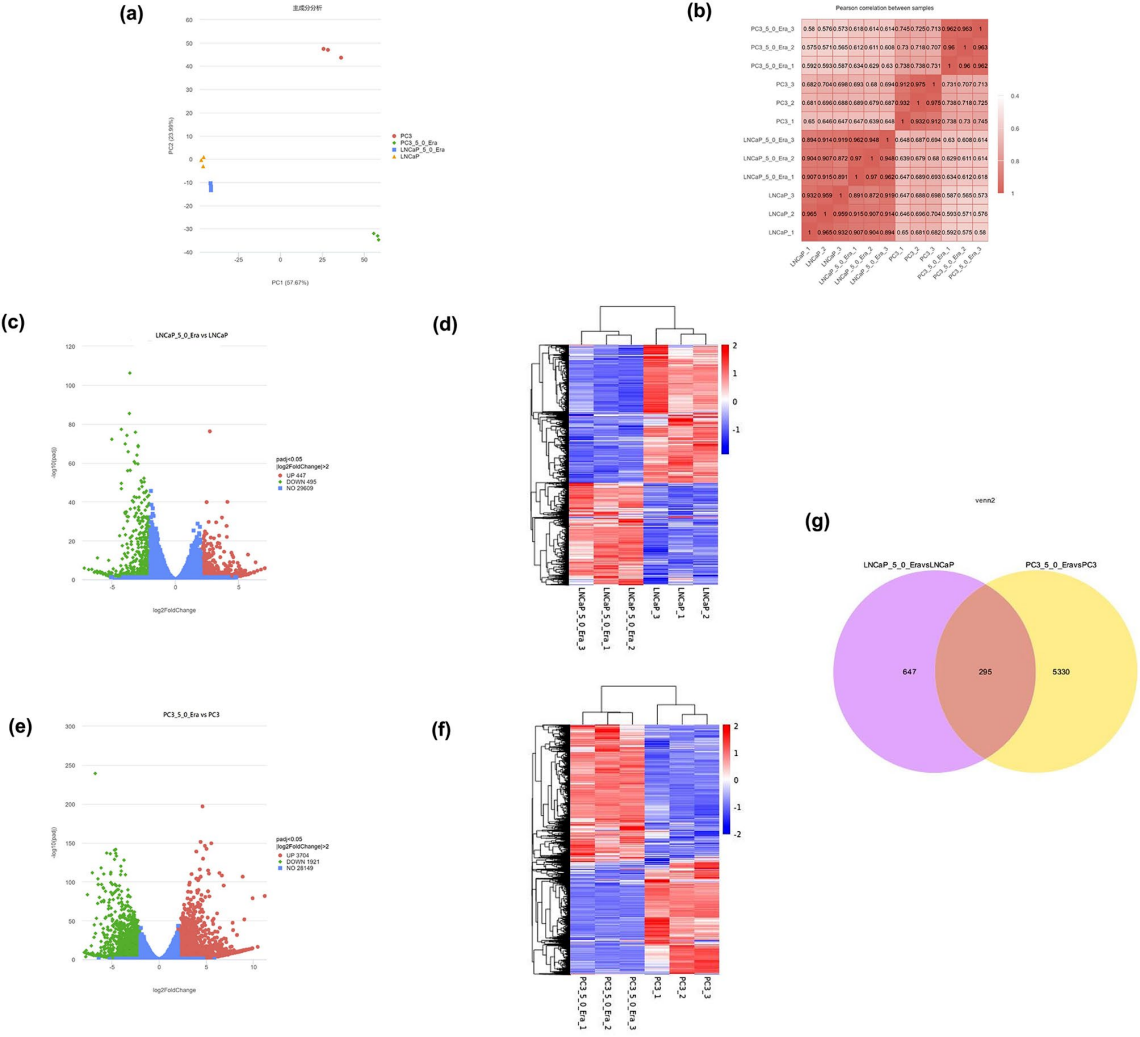
DEGs identification in LNCaP and PC3 cells after erastin exposure. (**a**) Principal component analysis (PCA) of RNA-seq data. (**b**) The correlation analysis results were consistent with PCA. (**c**) Volcano plot analysis identifies the DEGs of erastin-induced LNCaP groups; red and green dots represent 447 and 495 upregulated and downregulated genes, respectively. (**d**) Heatmap of LNCaP groups after erastin exposure. (**e**) Volcano plot analysis identifies the DEGs of PC3 groups; red and green dots represent 3,704 and 1,921 upregulated and downregulated genes, respectively. (**f**) Heatmap for PC3 groups after erastin exposure. (g) Venn diagram of erastin-induced DEGs

RNA sequencing was used to analyze the transcriptome profiling of PCa cells and erastin-induced PCa cells to investigate the molecular mechanisms by which the ferroptosis inducer erastin affects PCa cells and identify more FRGs. Using control cells and erastin-induced PCa cells, each of the four groups had three biological replicates, and 12 sequencing samples were prepared. Three replicates in four groups were consistent with the principal component analysis (Fig. 2a). Based on the correlation analysis results (Fig. 2b), the correlation coefficient of the samples was between 0.8 and 1, which showed good biological repeatability in these sequencing samples.

### Erastin-induced DEGs analysis

In the RNA-seq assay with padj ≤ 0.05 and |log2(foldchange)|≥2 as screening conditions, LNCaP_5_0_Era identified a total of 942 DEGs compared with LNCaP, including 447 upregulated genes and 495 downregulated genes (Fig. 2c–d). Then, a total of 5,625 DEGs were screened and recognized between PC3_5_0_Era and PC3, of which 3,704 were upregulated and 1,921 were downregulated (Fig. 2e–f). A total of 295 overlapping DEGs in the two compared groups were screened and recognized via a Venn diagram (Fig. 2g).

### Functional enrichment of erastin-induced genes and validation

**Fig. 3:**
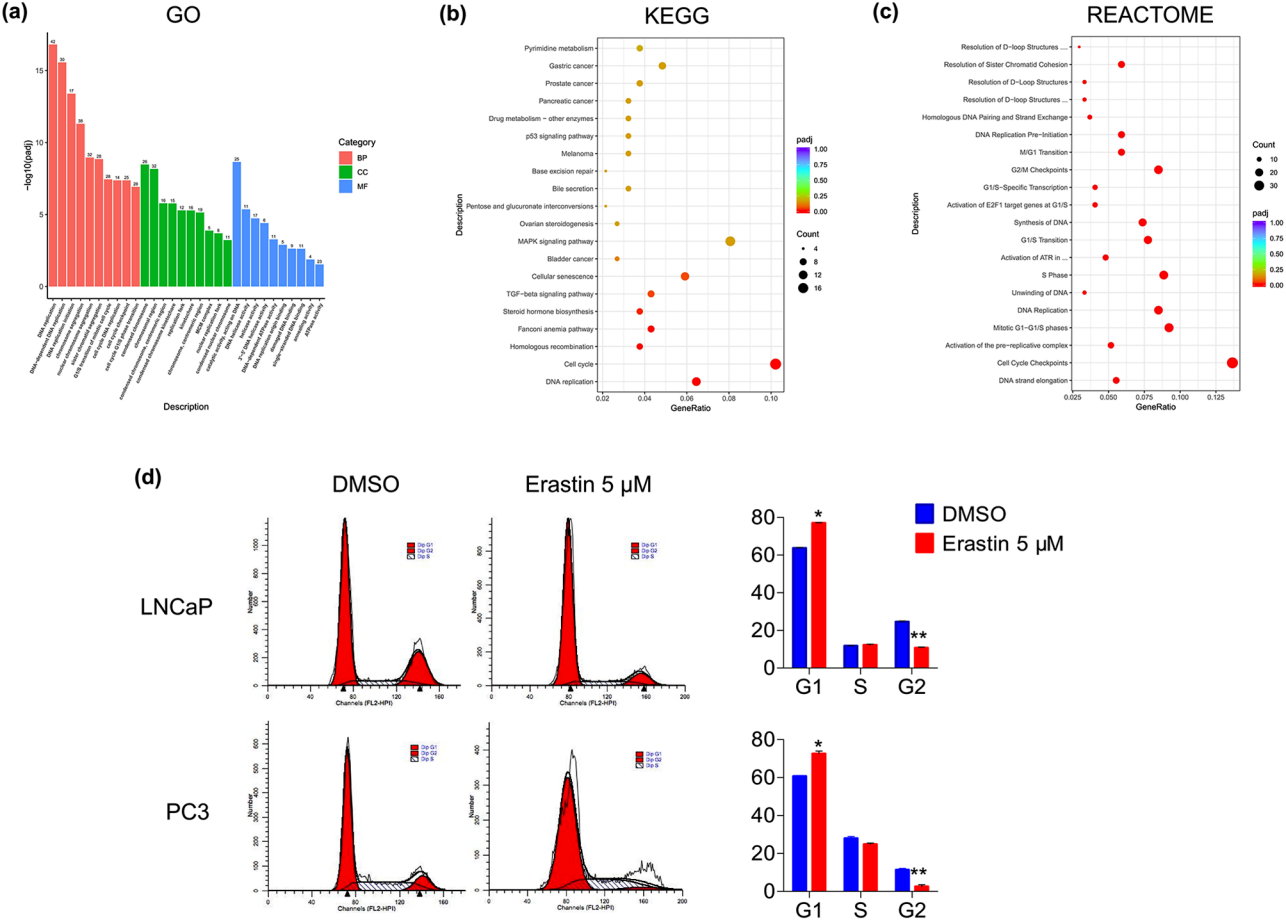
Enrichment analysis of the DEGs in erastin-induced LNCaP and PC3 cells. (**a**) GO functional analysis. BP, CC, and MF represent biological processes, cellular components, and molecular functions, respectively. (**b**) KEGG pathway analysis. (**c**) RECTOME functional analysis. (**d**) Flow cytometry detection of the cell cycle distribution of PCa cells after treatment with erastin. The results are expressed as a peak diagram, and the distributions of cells in the G1, S, and G2 phases were calculated. Statistical significance was calculated using an independent-sample t-test. *p < 0.05, **p < 0.01

GO, KEGG, and REACTOME functional enrichment analyses were performed to analyze 295 overlapping DEGs to further explore the potential biological behavior of erastin-induced changes in PCa cells. As exhibited by the GO analysis results, the erastin-induced DEGs were primarily related to DNA replication, DNA-dependent DNA replication, and DNA replication initiation for biological processes (BPs), condensed chromosome, chromosomal region, condensed chromosome, centromeric region for molecular functions (MFs), and catalytic activity acting on DNA, DNA helicase activity, and helicase activity for cellular components (CCs) (Fig. 3a). In contrast, KEGG analysis indicated that these DEGs mapped to DNA replication, cell cycle, and homologous recombination pathways (Fig. 3b). Furthermore, REACTOME analysis revealed that these DEGs were associated with DNA strand elongation, cell cycle checkpoints, activation of the prereplicative complex, and so on (Fig. 3c). To validate the functional enrichment analyses, the flow cytometry was employed to measure the cell cycle distribution. According to the flow cytometry modulation results, the percentage of PCa cells in G1 phase was considerably greater in the erastin-treated group than in the control group (*p* < 0.05; Fig. 3d).

### Pathways and module analysis of two PCa cells

An MCODE plug-in was carried out to construct modules representing the crucial clusters to access which related pathways contributed more weight to the ferroptotic process of PCa cells (Supplementary Fig. 1). Among the LNCaP group, the DEGs in module 1 were selected to conduct the next analysis, and they were found to be involved in several prominent signaling pathways (Supplementary Fig. 1a–b). For the PC3 group, enriched pathways, including nephron morphogenesis, positive regulation of blood vessel endothelial cell migration, positive regulation of bone mineralization, and regulation of steroid metabolic process, were detected in module 1 of PC3 DEGs (Supplementary Fig. 1c–d).

### GSEA analysis of erastin-induced genes

**Fig. 4:**
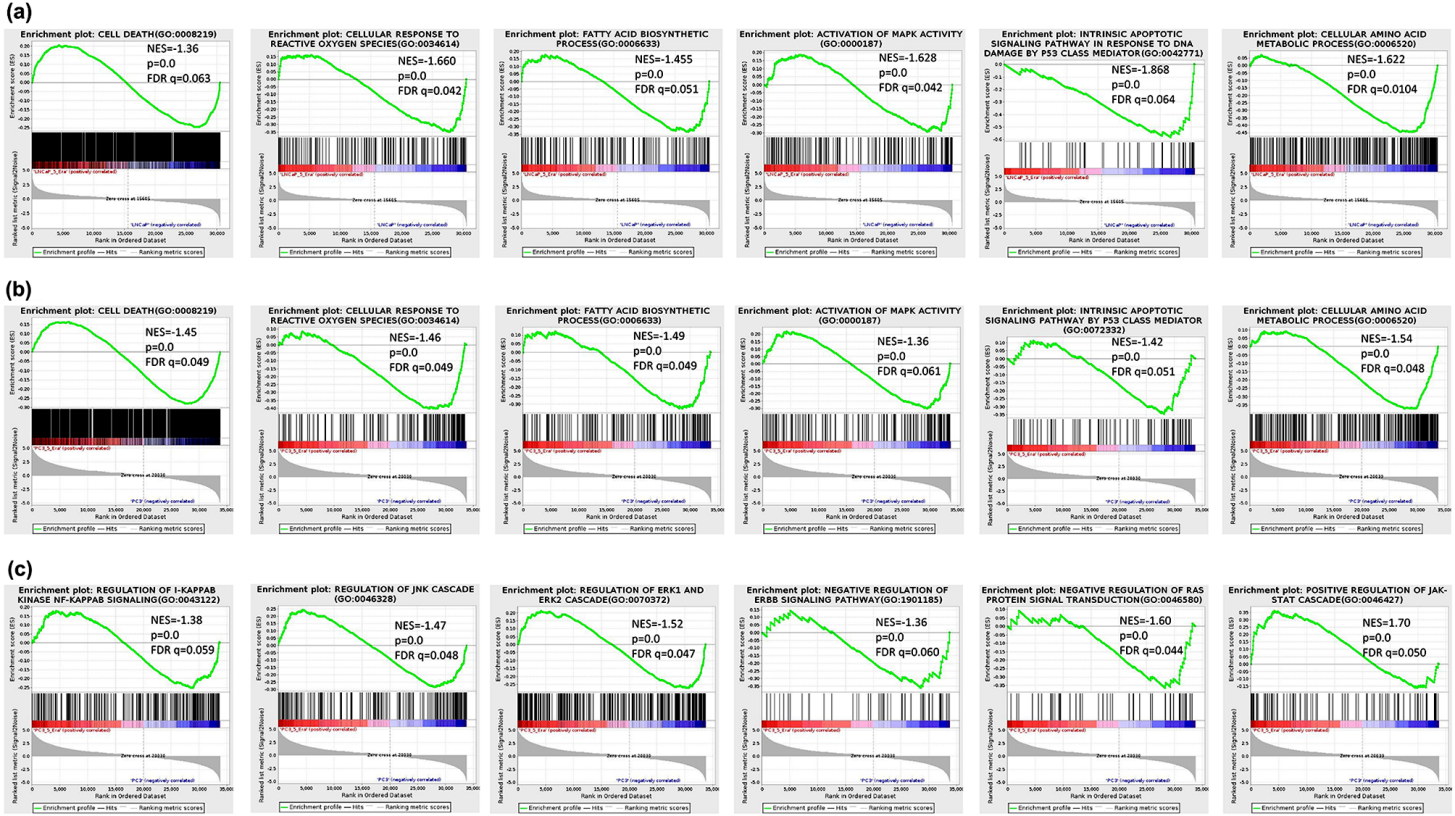
GSEA analysis based on the RNA-seq datasets. (**a**) GSEA analysis shows enrichment of the response genes in LNCaP group after erastin exposure. (**b**) GSEA analysis results of the enrichment of response genes in the PC3 group with erastin treatment. (**c**) GSEA analysis of enrichment of the response genes, which only appeared in the erastin-induced PC3 group. NES, normalized enrichment score; nominal *p*-value, and FDR, false discovery rate, and q-value were determined by GSEA software

Herein, we then performed GSEA analysis between the erastin treatment and control groups across the four cell lines to reveal more gene set enrichment information that involves the erastin-induced changes in PCa cells. We found that most of those pathways in GSEA were also found in the GO, KEGG, and Reactome functional enrichment analyses, which supports and validates the previous results. However, it was not unexpected to find that erastin-induced PCa DEGs were involved in the regulation of cell death, cellular response to reactive oxygen species, fatty acid biosynthetic process, activation of the MAPK activity pathway, intrinsic apoptotic signaling pathway in response to DNA damage by the P53 class mediator and cellular amino acid metabolic process in LNCaP (Fig. 4a) and PC3 cells (Fig. 4b). Moreover, we further focused on several pathways expected to be induced only in the PC3 group, including regulation of I-kappab kinase NF-kappab signaling, regulation of the JNK cascade, regulation of the ERK1 and ERK2 cascades, negative regulation of the ERBB signaling pathway, negative regulation of Ras protein signal transduction, and regulation of the JAK-STAT cascade (Fig. 4c).

### Erastin-induced hub genes and their TFs

**Fig. 5:**
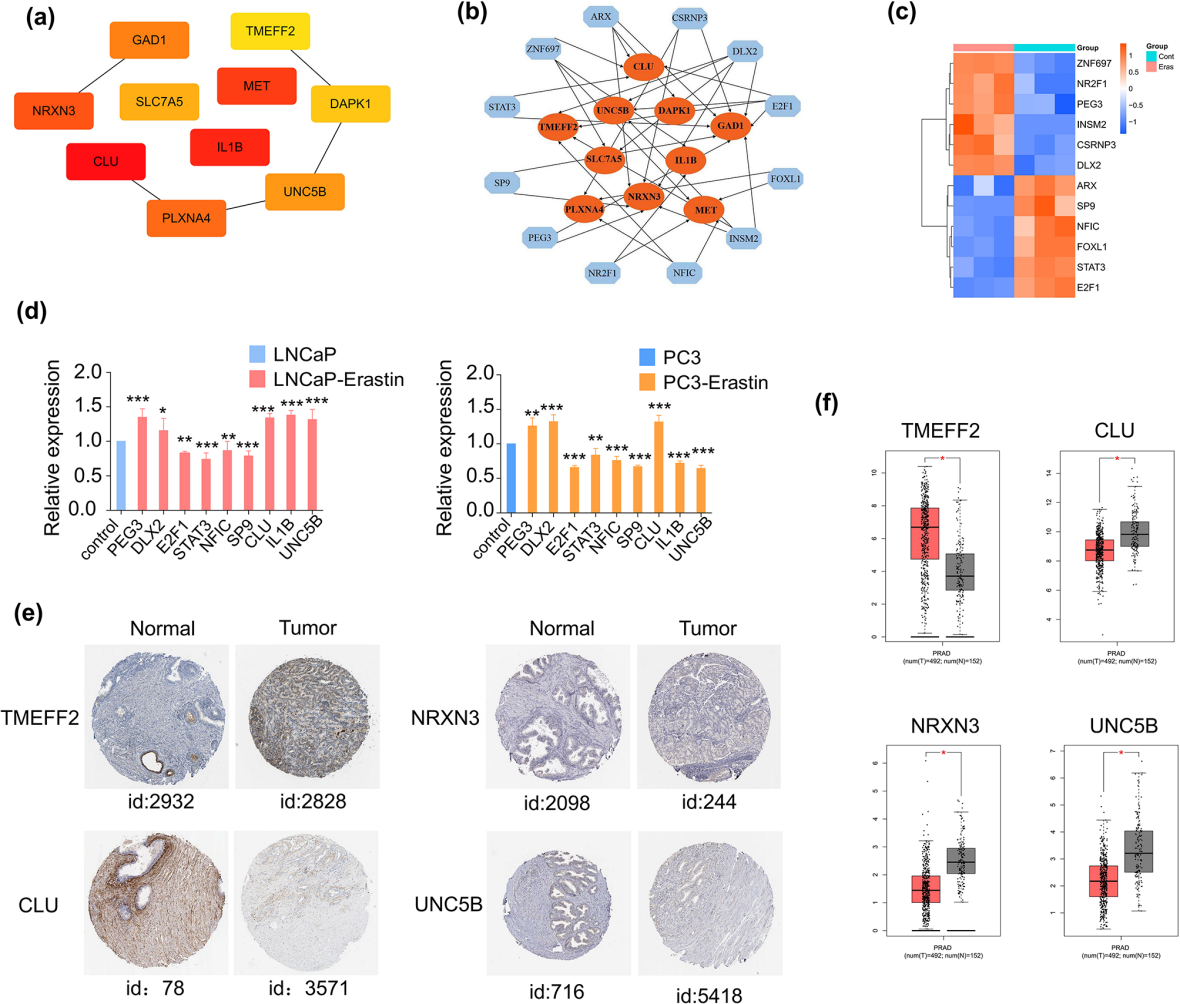
Prediction and validation the expression of TFs targeting hub genes and the clinical characteristics of the essential hub genes. (**a**) Top 10 hub DEGs of the LNCaP and PC3 cell groups with erastin treatment. (**b**) Interaction network of hub genes and their TFs. (**c**) The heatmap of the expression of the TFs was revealed by RNA-seq. (**d**) Expression of hub DEGs and TFs after erastin treatment were measured by qPCR, *n* = 3. The expression levels of *TMEFF2*, *CLU*, *NRXN3*, and *UNC5B* in normal tissues and prostate cancer tissue from the HPA database (**e**) and GEPIA database (**f**). *p < 0.05, *p < 0.01, ***p < 0.001

The cytoHubba plug-in was selected to calculate the hub genes of 295 overlapping DEGs to find more potential and pivotal genes related to ferroptosis in PCa cells. Of these, 10 top hub genes (including *CLU*, *IL1B*, *MET*, *NRXN3*, *PLXNA4*, *GAD1*, *UNC5B*, *SLC7A5*, *DAPK1*, and *TMEFF2*) were identified in order of degrees (Fig. 5a). Details and descriptions of the 10 hub genes are summarized in Table 1.Given that TFs had a critical influence on gene expression levels, the 10 top hub genes were uploaded to the NetworkAnalyst database for TF prediction. The regulatory network of the top TFs (including *SP9*, *DLX2*, *ARX*, *PEG3*, *CSRNP3*, *ZNF697*, *INSM2*, *STAT3*, *NR2F1*, *FOXL1*, *E2F1*, and *NFIC*) of the hub genes was obtained and presented (Fig. 5b). Moreover, the expression levels of these TFs are shown in Fig. 5c. Among them, *PEG3*, *CSRNP3*, *ZNF697*, *NR2F1*, *DLX2*, and *INSM2* were highly expressed, whereas *SP9*, *ARX*, *E2F1*, *FOXL1*, *STAT3*, and *NFIC* were expressed at low levels in PCa cells after erastin exposure. The expression levels of a few hub genes and TFs (including *CLU*, *IL1B*, *UNC5B*, *PEG3*, *DLX2*, *SP9*, *STAT3*, *E2F1*, and *NFIC*) were validated by qPCR (Fig. 5d). Moreover, we analyzed their expression patterns in the HPA and GEPIA databases to investigate whether these hub genes were clinically associated with PCa. In the HPA database, the expression of *TMEFF2* in PCa tissue was higher than that in normal tissue, and the expression levels of *CLU*, *NRXN3*, and *UNC5B* were lower in PCa tissue by immunohistochemistry (Fig. 5e). Consistently, *TMEFF2* mRNA levels were higher in PCa tissue (*p* < 0.05), and *CLU*, *NRXN3*, and *UNC5B* were downregulated (*p* < 0.05) in PCa tissue in the GEPIA database (Fig. 5f).

**Table 1:** Top 10 hub genes of erastin-induced LNCaP and PC3 cells

Ensemble ID	Symbol	log2(FC) In LNCaP group	FDR Value In LNCaP group	log2(FC) In PC3 group	FDR Value In PC3 group
ENSG00000120885	*CLU*	2.227676954	4.66194E-14	2.328754927	3.10046E-26
ENSG00000125538	*IL1B*	2.659181706	0.023399869	-2.354730765	5.37023E-07
ENSG00000105976	*MET*	2.178704253	5.57265E-06	-2.716782674	4.70167E-33
ENSG00000021645	*NRXN3*	2.219194193	2.12366E-07	2.442352118	6.87491E-18
ENSG00000221866	*PLXNA4*	3.383825988	4.60173E-05	5.0250723	0.006594555
ENSG00000128683	*GAD1*	2.674364639	5.49826E-07	4.298554233	1.7675E-116
ENSG00000107731	*UNC5B*	2.073123293	4.61178E-09	-2.989199096	3.58257E-30
ENSG00000103257	*SLC7A5*	-2.079560779	1.34346E-12	-2.392270174	1.17417E-23
ENSG00000196730	*DAPK1*	2.188978999	6.02751E-14	-5.892959015	8.06199E-77
ENSG00000144339	*TMEFF2*	-3.727660511	1.65524E-66	4.052271557	1.96873E-07

### Prediction and validation of FRGs

**Fig. 6:**
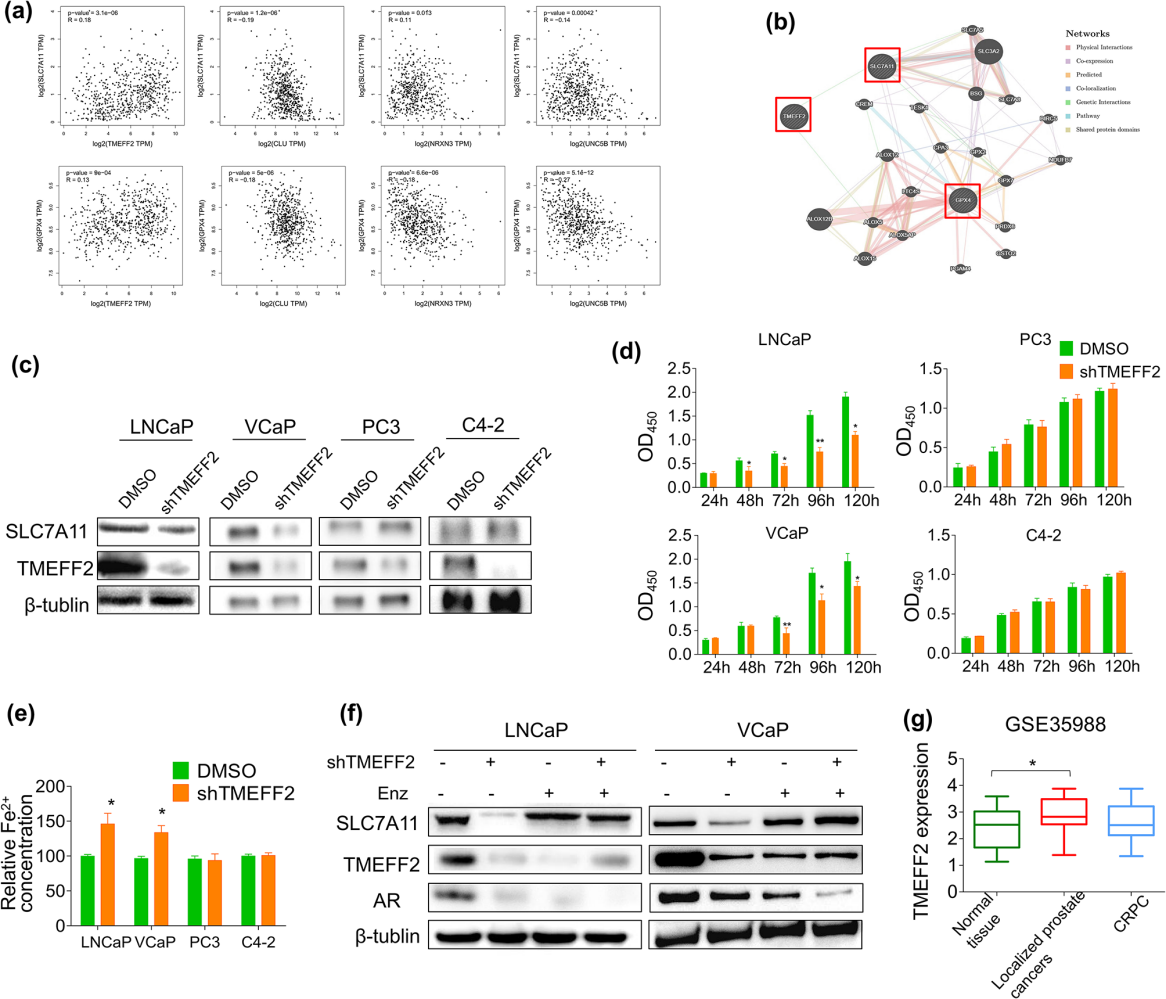
Correlation analysis and validation of FRGs. (**a**) Correlation analysis between four DEGs and *SLC7A11* and *GPX4*. (**b**) Gene co-expression network analysis of *TMEFF2*, *SLC7A11*, and *GPX4*. (**c**) The expression of *SLC7A11* was suppressed by downregulating *TMEFF2* in LNCaP and VCaP cells but not in PC3 and C4-2 cells. (**d**) The cell growth of LNCaP, VCaP, PC3 and C4-2 cells with knockdown of *TMEFF2* was examined via a CCK8 assay. Three independent experiments were performed with triplicate wells. Representative experiments and images are shown.(**e**) The relative Fe^2+^ concentration of LNCaP, VCaP, PC3 and C4-2 cells with downregulation of *TMEFF2* was detected. (**f**) After knockdown of *TMEFF2* and enzalutamide treatment, the expression of SLC7A11, TMEFF2, and androgen receptor (AR) were detected by western blot. (**g**) The mRNA expression of *TMEFF2* in normal tissue and prostate cancer tissue (including localized prostate tissue and CRPC tissue) was obtained from the GSE35988 dataset in the GEO database. The statistical significance of differences was calculated using an independent-sample t test. *p < 0.05, **p < 0.01

The relationships between hub gene expression levels and the ferroptosis markers *SLC7A11* and *GPX4* in the GEPIA database were analyzed to investigate whether these 4 hub genes were potentially involved in ferroptosis. As expected, the expression patterns of *TMEFF2*, *CLU*, *NRXN3*, and *UNC5B* were positively correlated with *SLC7A11* and *GPX4* (Fig. 6a). Gene interaction networks were established to clarify the possible direct relationship between hub genes and ferroptosis markers and identify their potential associations. *TMEFF2*, *SLC7A11*, and *GPX4* showed a complex PPI network with physical interactions, co-expression, prediction, co-localization, genetic interactions, pathways, and shared protein domains of 77.64%, 8.01%, 5.37%, 3.63%, 2.87%, 1.88%, and 0.60%, respectively (Fig. 6b). Among these networks, there were direct genetic interactions between *TMEFF2* and *SLC7A11*. Moreover, *TMEFF2* showed direct genetic interactions with *ALOX5*, and *ALOX5* showed physical interactions with *GPX4*. However, *CLU*, *NRXN3*, and *UNC5B* failed to display such a close relationship with *GPX4* compared with *TMEFF2* (data not shown). Therefore, *TMEFF* was selected as an interesting ferroptosis-related gene for further experiments.

In addition, we constructed LNCaP, VCaP, PC3 and C4-2 cells with *TMEFF2* downregulation to test whether *TMEFF2* can be an FRG in the experiment and then detected their cell survivability and Fe^2+^ concentration. Androgen-dependent LNCaP and VCaP cell lines with knockdown of *TMEFF2* exhibited lower expression of *SLC7A11*, while downregulation of *TMEFF2* did not significantly affect the expression level of *SLC7A11* in androgen-independent PC3 and C4-2 cell lines (Fig. 6c). After 48 h, the downregulation of *TMEFF2* significantly reduced the proliferative ability of LNCaP and VCaP cells (*p* < 0.05; Fig. 6d). After *TMEFF2* was knocked down, the level of Fe^2+^ in LNCaP and VCaP cells was markedly increased (*p* < 0.05; Fig. 6e). In contrast, there was no significant difference between PC3 and C4-2 cells with erastin treatment and the control group (Fig. 6e). To demonstrate the androgen-regulated property of *TMEFF2*, enzalutamide was selected to suppress the AR expression in LNCaP and VCaP cells. These cells were exposed to 10 µM enzalutamide for 48 h, which inhibited AR expression but not the expression of *SLC7A11* (Fig. 6f). LNCaP and VCaP cells with knockdown of *TMEFF2* and enzalutamide treatment, the expression of *SLC7A11* was not significantly different compared to cells with down-regulation of *TMEFF2* (Fig. 6f). Then, GSE35988 was utilized to confirm *TMEFF2* expression in clinical prostate tissue. Compared with normal tissues, the expression pattern of *TMEFF2* was markedly increased in localized prostate cancer. However, the *TMEFF2* expression levels did not significantly differ between normal tissues and CRPC tissues (Fig. 6g).

## Discussion

Our study discussed the role of the ferroptosis inducer erastin in two PCa cell lines, LNCaP and PC3. Unsurprisingly, we found that erastin exposure impacted LNCaP and PC3 cells in terms of ferroptosis markers, cell survivability, and the concentrations of MDA, Fe^2+^, GSH, and GSSG. In addition, we extracted the DEGs of both PCa cell lines at the transcriptional level. Subsequently, we identified various pathways, TFs and modules in different PCa cells. Moreover, we identified and validated several erastin-affected FRGs that were potentially meaningful in PCa treatment through a series of bioinformatics analyses and experiments.

First, we focused on the identification of FRGs in PCa cells after erastin exposure. From the RNA-Seq results, 10 FRGs were identified as hub genes by overlapping 295 DEGs. *TMEFF2*, a type I transmembrane protein with an EGF-like and two follistatin motifs 2 proteins, was selectively expressed in the brain and prostate [20]. Based on our analysis, *TMEFF2* was a co-expressed gene and correlated with *GPX4* and *SLC7A11*, as determined by GeneMANIA and GEPIA. Despite the absence of direct reports on the connection between *TMEFF2* and ferroptosis, a study reported that oxidative stress downregulates the expression of *TMEFF2* in PCa [21]. Moreover, *TMEFF2* can influence the proliferative activity of PCa cells [22], which was consistent with our results. In addition, similar to our results, several researchers reported that the alternative expression levels of *TMEFF2* changed the disease stage in PCa tissue of patients and a mouse model [23–25]. Therefore, the above reports supported our view that *TMEFF2* is related to ferroptosis and can be an FRG in PCa, and we first found that the expression level of *TMEFF2* was related to ferroptosis. It is worth mentioning that the ferroptotic level was significantly affected by knockdown of *TMEFF2* in LNCaP and VCaP cells but not in PC3 and C4-2 cells. LNCaP and VCaP cells with knockdown of *TMEFF2* and enzalutamide treatment, the expression of *SLC7A11* was not significantly different compared to cells with down-regulation of *TMEFF2.* LNCaP and VCaP are androgen-dependent cell lines and PC3 and C4-2 are androgen-independent cell line [11, 26]. Furthermore, *TMEFF2* has been identified as an androgen-related gene [22]. A report claimed that knockdown of *TMEFF2* could suppress the androgen response of LNCaP cells [27]. Moreover, we downloaded the GEO data and found that *TMEFF2* was highly expressed only in localized prostate cancer tissue and not in CRPC tissue. Thus, we speculated that *TMEFF2* promotes ferroptosis via androgen, which means that *TMEFF2* only influences ferroptosis in androgen-sensitive LNCaP cells. Therefore, if *TMEFF2* was used as a ferroptotic treatment target in the future, it would be more suitable for PCa patients than CRPC patients.

Our results also indicated that FRGs such as *NRXN3*, *CLU*, and *UNC5B* showed different expression levels between PCa and normal tissue, and their expression patterns were positively related to *GPX4* and *SLC7A11*. *NRXN3* has impacts on various functions of cancer, such as regulating the metastasis of nasopharyngeal carcinoma [28], inhibiting the proliferation and migration of glioma [29], and affecting the risk of breast cancer development [30]. Moreover, *NRXN3* is a gene associated with obesity [31], and we speculated that *NRXN3* participates in lipid metabolism. Our study was the first to reveal the differential expression of *NRXN3* between PCa tissue and normal tissue, and to examine the ferroptotic impact of PCa cells through the regulation of *NRXN3.* Furthermore, *CLU* causes ATP degradation, which results in peroxidation in cells [32], while the upregulation of *CLU* inhibits PCa cell death [33]. Moreover, *UNC5B* is a crucial regulator of ferroptosis in osteosarcoma cells [34]. Thus, the four hub DEGs identified in LNCaP and PC3 cells under erastin exposure play essential roles in the ferroptotic process in PCa cells and may be potential treatment targets for PCa.

Subsequently, we obtained a TFs network for the 10 DEGs in PCa cells after erastin treatment. Among the top TFs, *STAT3* and *E2F1* were differentially expressed in erastin-induced PCa cells, and accumulating evidence indicated that they are related to ferroptosis. *STAT3*, signal transduction and activators of transcription 3, can stimulates ferroptosis in PCa cells [35]. Mechanistically, *STAT3* binds to *GPX4* and regulates its expression in pancreatic cancer cells [36]. *E2F1*, E2F transcription factor 1, can be one of the ferroptosis-related gene prognostic indexes (FRGPI) to predict disease-free survival (DFS) for PCa patients undergoing radical prostatectomy. PCa patients with a high FRGPI had worse DFS than patients with a low FRGPI [37]. Hence, we speculated that these TFs associated with ferroptosis also participate in the regulation of hub gene expression to affect ferroptosis in PCa cells.

In addition to hub DEGs and their TFs, several pathways play an essential role in ferroptotic cells. Based on the GO functional analysis, the DEGs were primarily enriched in DNA replication, chromosome segregation, and the DNA helicase activity pathway. These three pathways are closely related to DNA replication, and DNA replication is the crucial link to cell growth. Our findings indicated that erastin markedly reduced the proliferative activity of PCa cells. In addition, Fe^2+^ is one of the essential factors of DNA replication, which is vital for cell survivability [38]. Consequently, we believed that erastin primarily transforms PCa cell proliferation by altering DNA replication. In addition to DNA replication, KEGG analysis revealed that these DEGs were also associated with steroid hormone biosynthesis, the MARK signal pathway, and P53 signal pathway. Steroid hormone biosynthesis is responsible for ferroptosis induction in adrenocortical carcinoma cells [39]. Considering our TFs network, it can be speculated that erastin affects MAPK/ERK to regulate *STAT3* and that *STAT3* is a TF that changes the expression patterns of the hub gene *CLU* in PCa cells. Furthermore, *STAT3* participates in the P53/SLC7A11 pathway in osteosarcoma cells [40]. In our results, *SLC7A11* was also downregulated by erastin exposure. We speculated that *STAT3* not only affects the expression level of *CLU* but also changes the P53 signal pathway to alter the expression of *SLC7A11*. Meanwhile, REACTOME enrichment analysis suggested that DEGs were enriched in cell cycle checkpoints and activation of E2F1 target genes in the G1/S pathway. In our study, erastin treatment significantly increased the percentage of PCa cells in the G1 phase. Moreover, cells in the G1 phase are strongly associated with the DNA replication process [41]. In addition, *E2F1* may be the TF of the hub gene *TMEFF2* according to our analysis; thus, *E2F1* could affect the transcript level of *TMEFF2*, which may be involved in the cell cycle of PCa cells. Therefore, erastin may affect the expression patterns of hub genes and their TFs by regulating various signaling pathways, including DNA replication, the cell cycle, and steroid hormone biosynthesis, to transform the cell growth and cell cycle of PCa cells.

Interestingly, we found that the erastin-induced PC3 group had more DEGs than the erastin-induced LNCaP group. In our module analysis, the top 1 module of the PC3 group had more direct ferroptosis-related pathways, such as positive regulation of bone mineralization and regulation of steroid metabolic process, than the LNCaP groups. Therefore, for these two PCa cell lines, we suggested that PC3 cells are much more sensitive to the ferroptosis inducer erastin than LNCaP cells. Subsequently, we wanted to know why the CRPC cell line PC3 exhibited more susceptibility to erastin. GSEA was applied to study various cellular signaling pathways that other functional enrichments did not identify. As expected, several cell death-related pathways were enriched, and the PC3 group displayed an increasing number of unique signaling pathways in GSEA, such as negative regulation of the ERBB signaling pathway, negative regulation of Ras protein signal transduction, and regulation of the JAK-STAT cascade. These signaling pathways are all associated with ferroptosis in various cells. A complex suppressed the ERBB signal pathway to inhibit ferroptosis in vivo and in vitro [42]. Moreover, the RAS/MAPK pathway may regulate oxidative stress and the related ferroptosis pathways in glioblastoma [43]. Furthermore, IFNγ enhanced the JAK-STAT signal pathway to activate ferroptosis in hepatocellular carcinoma cells [44]. Unfortunately, there is a lack of direct reports about the relationship between these signaling pathways and ferroptosis in PCa. Hence, erastin transformed more crucial signaling pathways, which led to PC3 being more sensitive to erastin. Moreover, considering that PC3 is an androgen-independent cell line and LNCaP is an androgen-sensitive cell line [45], we speculated that CRPC patients may be more susceptible to targeted gene therapy related to ferroptosis than PCa patients.

This current study has some limitations. First, two PCa cell lines, LNCaP and PC3, were selected for the experiment. If we included more PCa cells, the results would be more comprehensive. Second, although we verified the FRGs in cell experiments and in public databases, our further study will be confirmed in animal experiments and clinical practice.

## Conclusions

This study verified that erastin successfully brought about a significant effect on the proliferation and ferroptotic index of LNCaP and PC3 cells. In addition, we carried out RNA-seq and annotated 295 overlapping DEGs after erastin treatment in these two cell lines. Functional enrichment analysis showed that erastin may act on PCa cells by participating in a variety of pathways, including DNA replication and the cell cycle. The G1 phase distribution of LNCaP and PC3 cells treated with erastin was obviously increased. Our results also indicated that several FRGs, such as *TMEFF2*, *NRXN3*, *CLU*, and *UNC5B*, showed different expression levels between PCa and normal tissue. The potential TFs *STAT3* and *E2F1* may take part in regulating the ferroptotic process of these FRGs. We also found that downregulation of *TMEFF2* only promote ferroptosis in androgen-sensitive LNCaP and VCaP cells but not in androgen-independent PC3 and C4-2 cells. Compared with normal tissues, the expression pattern of *TMEFF2* was markedly increased in localized prostate cancer, and but not obviously upregulated in CRPC tissues. Our findings elucidate potential molecular mechanisms in subsequent ferroptotic studies of PCa cells and suggest novel therapeutic targets and strategies for PCa.

### Electronic supplementary material

Below is the link to the electronic supplementary material.

Supplementary Material 1

Supplementary Material 2

Supplementary Material 3

## Data Availability

The original contributions presented in the study are publicly available. This data can be found here: GSE232034 datasets downloaded from the GEO database.
