# Supplementary figures and images for: Identification of ferroptosis related genes and pathways in prostate cancer cells under erastin exposure
Source: BMC Urol. 2024 Apr 4;24:78. doi: 10.1186/s12894-024-01472-1 (PMC10996193; doi:10.1186/s12894-024-01472-1)

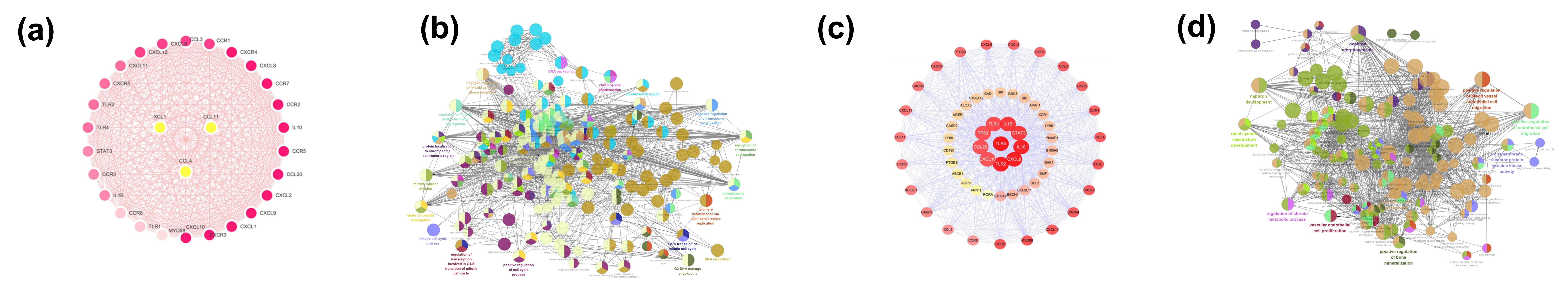

Supplement: Supplementary file 1 — Supplementary Material 1 [file 12894_2024_1472_MOESM1_ESM.tif]
