# Supplementary material for: Identification of ferroptosis related genes and pathways in prostate cancer cells under erastin exposure
Source: BMC Urol. 2024 Apr 4;24:78. doi: 10.1186/s12894-024-01472-1 (PMC10996193; doi:10.1186/s12894-024-01472-1)
Supplement: Supplementary file 2 — Supplementary Material 2 [file 12894_2024_1472_MOESM2_ESM.docx]

**Supplementary Fig. 1** Module and enrichment analysis of LNCaP and PC3 cells after erastin exposure. (a) A cross-link plot of the significant functional modules in the LNCaP group was constructed with the MCODE plug-in. (b) The DEGs in the module and their enriched pathways defined by ClueGO in the LNCaP group. (c) The module plot of significant functional categories in the PC3 group was constructed with the MCODE plug-in. (d) The DEGs in the module and their enriched pathways defined by ClueGO in the PC3 group.
