# Supplementary material for: Identification of ferroptosis related genes and pathways in prostate cancer cells under erastin exposure
Source: BMC Urol. 2024 Apr 4;24:78. doi: 10.1186/s12894-024-01472-1 (PMC10996193; doi:10.1186/s12894-024-01472-1)
Supplement: Supplementary file 3 — Supplementary Material 3 [file 12894_2024_1472_MOESM3_ESM.docx]

**Supplementary Table 1** **Primers for qPCR in this study**

| Primer Name | Sequence - 5´- 3´ |
| --- | --- |
| GAPDH-F | GCACCGTCAAGGCTGAGAAC |
| GAPDH-R | TGGTGAAGACGCCAGTGGA |
| CLU-F | GGTGTGTGCGCGAGCAGAG |
| CLU-R | GCCTCGGTCAGCGGCACC |
| IL1B-F | CCACAGACCTTCCAGGAGAATG |
| IL1B-R | GTGCAGTTCAGTGATCGTACAGG |
| UNC5B-F | GAGCCGAAACCGCTAATG |
| UNC5B-R | CTGCCACTCCAAATGTGATAGA |
| PEG3-F | AACCTCTGCCTCGATTGGTT |
| PEG3-R | GTCCCGGTCACTGAAAGAATG |
| DLX2 -F | CATGGGCTCCTACCAGTACCAC |
| DLX2 -R | TCGGATTTCAGGCTCAAGGTC |
| E2F1-F | GCCACTGACTCTGCCACCATAG |
| E2F1-R | CTGCCCATCCGGGACAAC |
| STAT3 -F | CAGAAAGTGTCCTACAAGGGCG |
| STAT3 -R | CGTTGTTAGACTCCTCCATGTTC |
| NFIC-F | TGGCGGCGATTACTACACTTCG |
| NFIC-R | GGCTGTTGAATGGTGACTTGTCC |
| SP9-F | TCTATACTCGGGGAAGAGCCG |
| SP9-R | GAAGGCGCTCGACTCTGG |
